# Supplementary figures and images for: A RT-qPCR system using a degenerate probe for specific identification and differentiation of SARS-CoV-2 Omicron (B.1.1.529) variants of concern
Source: PLoS One. 2022 Oct 5;17(10):e0274889. doi: 10.1371/journal.pone.0274889 (PMC9534396; doi:10.1371/journal.pone.0274889)

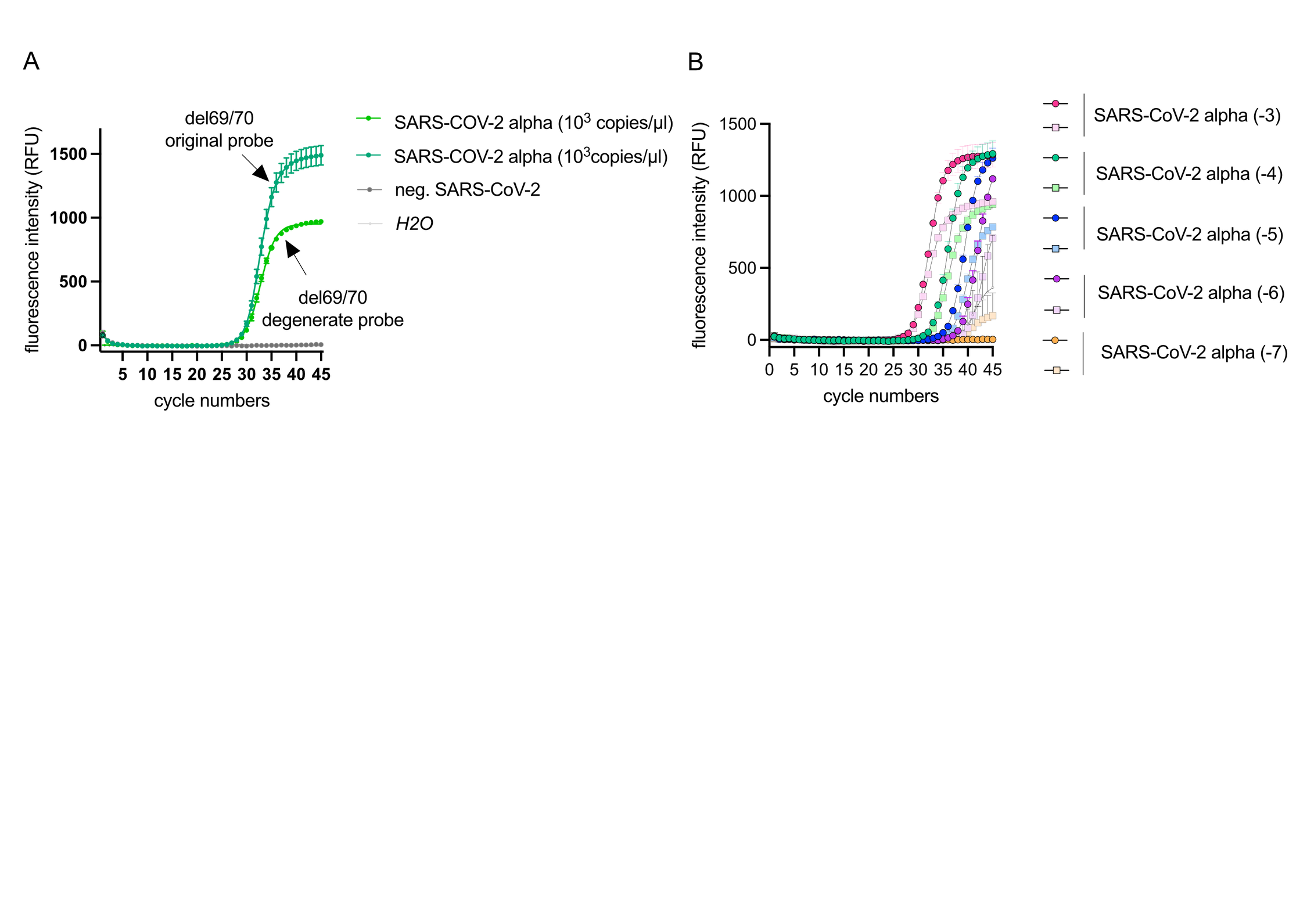

Supplement: S1 Fig — (A) Dilution of the TWIST control standard (SARS-CoV-2 Alpha variant) to a concentration of 10−3 copies/μl detected by the original and the degenerate probe targeting the ΔH69/V70. (B) Dilution row of the TWIST control standard (SARS-CoV-2 Alpha variant) detected by the original and the degenerate probe targeting the ΔH69/V70. (TIF) [file pone.0274889.s001.tif]
